# Immunoglobulin A response to SARS-CoV-2 infection and immunity

**DOI:** 10.1016/j.heliyon.2024.e24031

**Published:** 2024-01-03

**Authors:** Khaleqsefat Esmat, Baban Jamil, Ramiar Kaml Kheder, Arnaud John Kombe Kombe, Weihong Zeng, Huan Ma, Tengchuan Jin

**Affiliations:** a Department of Obstetrics and Gynecology, The First Affiliated Hospital of USTC, Center for Advanced Interdisciplinary Science and Biomedicine of IHM, Division of Life Sciences and Medicine, University of Science and Technology of China, Hefei, Anhui, 230001, China; b Department of Medical Analysis, Faculty of Applied Science, Tishk International University, KRG, Erbil, Iraq; c Medical Laboratory Science Department, College of Science, University of Raparin, Rania, Sulaymaniyah, Iraq; d Laboratory of Structural Immunology, School of Basic Medical Sciences, Division of Life Sciences and Medicine, University of Science & Technology of China, Hefei, Anhui, 230027, China; e Institute of Health and Medicine, Hefei Comprehensive National Science Center, Hefei, Anhui, China

**Keywords:** SARS-CoV-2, COVID-19, Immunoglobulin A (IgA), Immune response, Serological test, Mucosal immunity, Vaccine

## Abstract

The novel coronavirus disease (COVID-19) and its infamous “Variants” of the etiological agent termed Severe Acute Respiratory Syndrome Corona Virus 2 (SARS-CoV-2) has proven to be a global health concern. The three antibodies, IgA, IgM, and IgG, perform their dedicated role as main workhorses of the host adaptive immune system in virus neutralization. Immunoglobulin-A (IgA), also known as “Mucosal Immunoglobulin”, has been under keen interest throughout the viral infection cycle. Its importance lies because IgA is predominant mucosal antibody and SARS family viruses primarily infect the mucosal surfaces of human respiratory tract. Therefore, IgA can be considered a diagnostic and prognostic marker and an active infection biomarker for SARS CoV-2 infection. Along with molecular analyses, serological tests, including IgA detection tests, are gaining ground in application as an early detectable marker and as a minimally invasive detection strategy. In the current review, it was emphasized the role of IgA response in diagnosis, host defense strategies, treatment, and prevention of SARS-CoV-2 infection. The data analysis was performed through almost 100 published peer-reviewed research reports and comprehended the importance of IgA in antiviral immunity against SARS-CoV-2 and other related respiratory viruses. Taken together, it is concluded that secretory IgA- Abs can serve as a promising detection tool for respiratory viral diagnosis and treatment parallel to IgG-based therapeutics and diagnostics. Vaccine candidates that target and trigger mucosal immune response may also be employed in future dimensions of research against other respiratory viruses.

## Abbreviations

ACE2: Angiotensin-converting enzyme 2
ADE: antibody-dependent enhancement
CDR: complementarity determining regions
CP: convalescent plasma
ELIZA: (enzyme-linked immunosorbent assay)
HIV: human immunodeficiency virus
HCoVs: Human CoVs
ICTs: Immuno Chromatographic tests
Igs: immunoglobulins
IgA: immunoglobulin A
LAMP: Loop-Mediated Isothermal Amplification
MAbs: monoclonal antibodies
MERS-CoV: Middle East Respiratory Syndrome-related coronavirus
nAbs: neutralizing antibodies
NCBI: National Center for Biotechnology Information
PCR: polymerase chain reaction
POC: point of care
PSO: post-symptom onset
pIgA: polymeric immunoglobulins A
qRT-PCR: Quantitative Reverse Transcription PCR
RBD: receptor-binding domain
RNA: Ribonucleic acid
RLU: relative light units
SARS-CoV: severe acute respiratory syndrome-related coronavirus
SC: secretory component
SIgA: Secretory IgA

## 1 Introduction

Coronaviruses (CoVs), as a vast group of positive single-stranded RNA (+ssRNA) viruses, affect humans and, in some cases, animals. Human CoVs (hCoVs) are mainly associated with upper respiratory tract diseases such as common cold and bronchiolitis. The severe acute respiratory syndrome (SARS)-CoV-2 infects humans in the lower respiratory tract via airway epithelial cells and appears to be the most readily contagious virus among the known hCoVs [1,2]. Convalescent plasma (CP) due to high titer antibodies and neutralization capacity, and immunization by vaccines were effective way to combat COVID-19 infection. Molecular tests for SARS-CoV-2 diagnosis are considered as gold standard for diagnosis of an active infection, they are limited by low sensitivity which results in high false-negative result rate in case of slight contamination or even a simple pipetting error. Besides, the high costs of instruments, the sampling-associated difficulties, and the time-consuming phase of nucleic acid extraction for quantitative reverse transcription PCR (qRT-PCR) still pose a hurdle in developing quick, point of care (POC) molecular diagnostic tests. This qRT-PCR should not be confused with Loop-Mediated Isothermal Amplification (LAMP), strictly considered a “Screening” test for rapid COVID screening. Therefore, as a POC, Immuno Chromatographic tests (ICTs) (Either detecting viral antigen or an antibody against the virus) are employed internationally. With guidelines differing state by state, the fundamental principle remains the same, i.e., either qRT-PCR for SARS CoV-2 RNA from nasopharyngeal or oropharyngeal swabs and/or ICR for SARS-CoV-2 antigen/antibody or quantitative enzyme-linked immunosorbent assay (ELISA) to determine immune response by checking antigen or antibody titer in patient's samsple (serum/plasma/blood). All mentioned techniques have different efficiencies, specificity, and sensitivity [[3], [4], [5]]. Several studies support that serological tests can detect SARS-CoV-2-specific antibodies with high specificity and are sometimes more reliable than molecular diagnostic tools [[6], [7], [8], [9]]. SARS-CoV-2 serological tests based on the detection of immunoglobulins (Igs) against SARS-CoV-2 spike protein (S-protein) and nucleocapsid (N-protein) are among the most widely used ones [[10], [11], [12]].

Interestingly, IgA is the main isotype target that is specifically produced upon SARS-CoV-2 infection and transported by respiratory epithelium, and therefore, due to its titer in the patient's mucosal lining makes its concentration highly attractive target as the accuracy of the immunodiagnostic tests comes under the limit of detection (LOD) of a serological test [6]. The secreted IgA (sIgA) is central to mucosal immunity, which attacks the infectious pathogens in the respiratory and digestive system's entrance, thereby neutralizing viruses or impeding their attachment to epithelial cells [13]. The intranasal immunization intervention of the Middle East Respiratory Syndrome-related coronavirus (MERS-CoV) in responses to the derived vaccine has confirmed IgA's beneficial role [14]. Intranasal inoculation of vaccine SARS-CoV in animal models caused localized virus-specific IgA secretions and subsequent immune response, providing better protection against SARS-CoV than intramuscular delivery, suggesting mucosal-induced immunity can provide proof that a SARS-CoV vaccine is feasible [15]. The receptor-binding domain (RBD) specific IgA in respiratory mucosa might be considered an early indicator of host immunological response, which can be measured from various external secretion samples and might be employed for further treatment against SARS-CoV-2 [[16], [17], [18], [19], [20]]. Previous studies on influenza and HIV-specific antibodies have shown similar observations [21]. Although the effectiveness of the IgA antibody isotype as a potential neutralizing monoclonal antibody in mitigating COVID-19 already have reported, but its exact molecular mechanism of action, particularly against SARS-CoV-2 remains unclear [[22], [23], [24], [25], [26]]. It was investigated that wild-type SARS-CoV-2 spike-specific mucosal IgA is protective against COVID-19 and omicron virus infection [27,28] and also against SARS-CoV-2 and vaccination [27]^.^ It was investigated that only IgA immunoglobulin is correlated corelated with critical COVID-19 disease [29] and IgA immunoglobulin elevation during vaccination can be protective for the infection of SARS-CoV-2 virus disease [30]. Our review will investigate the specific role of IgA in SARS-CoV-2 virus infection, its importance, and its possible mechanisms of action and to combat the COVID-19 virus infection.

### 1.1 IgA structure and its secretion component

Human IgA possesses two heavy chain constant (Hc) regions with two isotypes termed IgA1 and IgA2. Among these two isotypes, IgA1 is distinguished with wide-open hinges that markedly contrast the standard “Y” shaped immunoglobulin structure. Species other than higher primates do not have the IgA1 isotype [[31], [32], [33], [34]]. This structure of monomeric IgA is synonymous with other immunoglobulins classes. In the case of this H2L2 monomeric unit can polymerize further [35]. In dimeric IgA, two monomer units are arranged in an end-to-end configuration stabilized by disulfide bridges and incorporating the joining chain (JC), a 15 kDa polypeptide [36]. Therefore, IgA's polymerization requires a homologs protein structure consisting of 137-residues JC [37]. The joining (J chain) is synthesized along with IgA in plasma cells, and its incorporation is an early event in IgA polymerization. J-chain contributes to IgA dimerization, and the secretory component (SC) protects this dimer from the microenvironment, proteolysis, and hydrolysis, thus stabilizing the overall dimer. Consequently, this peptide is found in all polymeric forms of IgA [38]. IgA in secretions exists in dimeric and tetrameric forms while approximately 60 % occurs in the dimeric form, 40 % presents in tetrameric form as documented in human milk and saliva [39]. The secretory component is secreted in the mucosal lining and prone to deviations in the microenvironment, thus protecting the IgA dimer. The secretory component of IgA is pivotal for its optimal activity, also most required in gut lining for dimer protection. In the respiratory tract, the secretory components assist IgA dimers to localize on the mucosal lining, especially in the alveolar area of the lungs [40]. Mucosal plasma cells synthesized polymeric immunoglobulins A (pIgA) produces their protective activities by transcytosis to reach the external secretions. Initiation of this process requires the pIgA binding to the ectodomain of basolateral expressing receptor pIg (pIgR), and the JC can stabilize such interaction through a disulfide bond joining the receptor and one Fc [41].

**Fig. 1:**
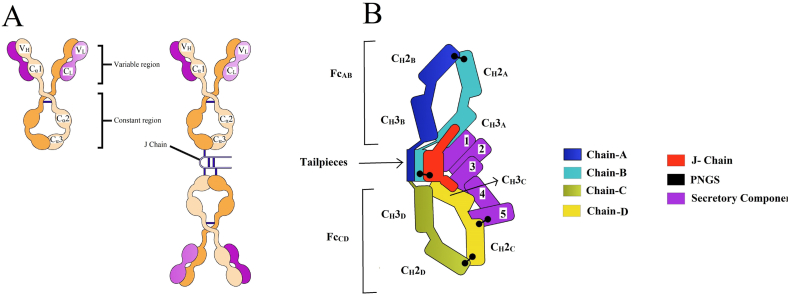
Monomeric and dimeric IgA. A) Left: The monomeric IgA (mIgA). The heavy chains are shown in orange and the light chains in purple. V: Variable region, C: Constant region. Right: The dimeric IgA (dIgA). dIgA contains the joining chain (J-CHAIN) linked by two disulfide bonds to the Fc region in the two different monomers. B) Schematic representation of the complex structure of SIgA with the secretory component. Chain names and corresponding C_H_ domains and Fcs are labeled with SC domains (1–5). Each SIgA component is depicted in a unique color. This figure is based on recently published papers [46]. (For interpretation of the references to color in this figure legend, the reader is referred to the Web version of this article.)

After binding to the pIgR, pIgA is transported to mucosa through transcytosis of epithelial cells. After transcytosis, anonymous proteases digestion causes the release of secreted components of the pIgR, and the proteases stay covalently with pIgA to form secretory IgA, which performs antimicrobial neutralization functions [42,43]. sIgA is a tail-to-tail planar dimer (at ∼110° angle) with two Fc regions holding positions by the J chain, causing clasp function. The secretory form is extensive for contiguity with Fcs, and J-chain is diagonally bound between the two monomers across the ∼50° gap. Incorporation of two or another three Fcs in-plane to the native dimer assembles higher-order polymers. Five Ig-like domains (D1 to D5) comprised by SC, D1 is needed and adequate for pIgA binding, although D2 to D5 provide affinity enhancement [44] the lack of PIgA results in the closing SC adopts distinct conformation by the interaction among D1, D4, and D5 [45] (Fig. 1(A and B)).

Previous structural studies revealed that the SC underwent a large conformational change and interacted through both domains of D1 and D5 together with both Fcs of the IgA dimer core and the J-chain. The asymmetry imparted by the J-CHAIN allows the dimer's one-to-one binding of the asymmetric Secretory Chain. The interaction is noncovalent and involves all three Complementarity Determining Regions (CDR)-like loops with D1, whereas D5 interaction is mediated by a single disulfide bond [47].

Although D2 to D4 have no direct contact with pIgA, they assist correct spacing to allow D1 and D5 to bind to the dimer. D2 to D5 are arranged in a head-to-tail manner. D1 positioning allows its three CDRs to bind pIgA through a sharp 180° turn in the D1–D2 linker. An aromatic-rich D1–D3 interface stabilized the described kinked SC conformation. The buried surface area of approximately one-third of the ∼10–55 Å of D1 results from interactions with the C terminus of J-CHAIN. This entire assembly is consistent with the J-CHAIN requirement for pIgR binding transcytosis [36,46] (Fig. 1(A and B)).

### 1.2 Role of IgA in antiviral immunity

IgA is present in serum, nasal mucus, saliva, breast milk, and intestinal ﬂuid, making IgA-containing sample collection easy [48]. Three defense functions are designated for IgA, namely, immune exclusion, intracellular neutralization and viral excretion or viral clearance. IgA after secretion can bind microbial surfaces and thus avoid their attachment or penetration to the epithelial layers, although its mechanism is not fully understood yet. Research data on the influenza virus propose that the IgA neutralization mechanism may vary depending on the molecular weight of the virion or surface in contact's size or epitope length [49].

IgA is supposed to interact with intracellular pathogens, including viruses, through the lining epithelial cells after polymeric immunoglobulin receptor (pIgR)-mediated endocytosis [13]. Such intra-epithelial cell neutralization was demonstrated by IgA monoclonal antibodies (MAbs) against the Influenza virus, Sendai virus, and rotavirus [50,51]. In mice, an IgA mAb toward a rotavirus internal protein could avert infection and cure persistent infection [52]. The dimeric secretory form of IgA had in mucosa is over one log more potent than the monomer against authentic SARS-CoV-2 which initiates neutralizer more than IgG [53]^.^ The neutralizing antibodies received from convalescent patients or vaccinees could have antibody-dependent enhancement activities against SARS-CoV2 variants [54].^.^

IgA in the lamina propria beneath mucosal epithelium may form a complex with antigens and transport them via the pIgR across the epithelial cells into the secretions [55,56]. Epstein-Barr virus-IgA immune complexes were transcytosed across polarized epithelial cells from the basolateral to the apical surface [57]. In a variant of this excretory immune function, when IgA antibodies interacted with free human immunodeficiency virus (HIV) virion particles within epithelial cells, the antibodies blocked their apical-to basal-surface transcytosis transporting viral particles to the apical supernatant [58].

### 1.3 Importance of IgA in SARS-CoV-2 diagnosis and immunization

SARS-CoV-2 serological tests were planned to distinguish various antibody isotypes present after virus infection [59]. The S-protein and N-protein of SARS-CoV-2 have critical antigenic sites to improve serological assays [[60], [61], [62]]. Serological tests for detecting anti-SARS-CoV-2 antibodies in a population allow the identification of people who have acquired immunity against SARS-CoV-2 and describe the seroprevalence of SARS-CoV-2 infection. Currently, there are many serological tests, including manual and automated tests, developed to detect SARS-CoV-2-specific antibodies in the patient's samples; however, there is variability in their specificity and sensitivity [63].

At the beginning of the COVID-19 outbreak in early 2020, during purification of SARS-CoV-2 S-protein receptor-binding domain (RBD) and N-protein specific antibodies from a serum pool of hospitalized patients, it was found the increase levels of IgA antibody in addition to expected IgM and IgG immunoglobulins [[60], [61], [62]]. Thus, it was developed chemical luminescent diagnostic kits of IgA in addition to traditional IgM/IgG kits against RBD of SARS-CoV-2 and validated the effectiveness in early diagnosis of COVID-19. It was demonstrated for the first time that by combining IgA/IgM/IgG, the diagnostic accuracy improved significantly, and IgA can be used as an early marker for COVID-19 [20,64].

In the meanwhile, other reports verified the findings and demonstrated that IgA antibody can be a suitable marker for antigen/antibody-based COVID-19 diagnosis [[60], [61], [62]]. The SARS-CoV-2 infection is followed by an earlier and strong IgA antibody response because, by nature, SARS-CoV-2 targets ACE receptors, constitutively expressed throughout respiratory tract mucosal cells. IgA pools are predominantly abundant crypt cells and active mucosal immune systems in the mucosal lining that rapidly capture virions and transfer them to nearby lymph nodes where germinal centers start producing Igs. The B cells are then switched under the influence of local chemokines and cytokines to class switch the initial IgM to IgA, which is rapidly released in the gut mucosa. Detection of SARS-CoV-2 antibodies, especially IgA produced in the early days of post symptom onset (PSO) combined with PCR, can improve the prevalence of infection sensitivity and accuracy [65].

### 1.4 Antibody kinetics in SARS-CoV-2 infection

The five isotypes of human antibodies with well-defined characteristics and roles based on their H chains are IgM, IgD, IgG, IgA, and IgE [66,67]. However, only IgM, IgG, and IgA are commonly used in serological testing for antigen identification.

**Fig. 2:**
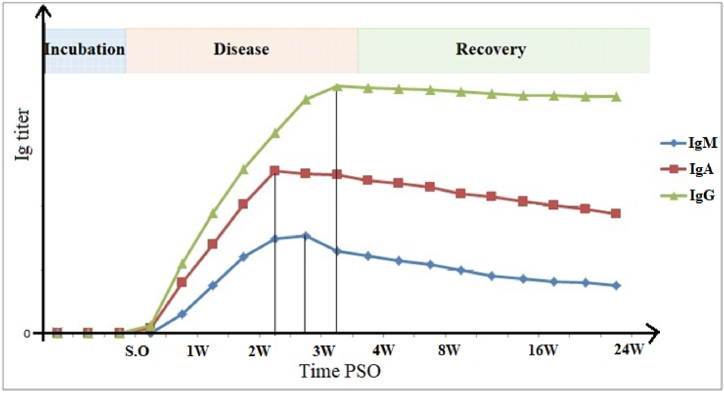
Schematic representation of serum antibody kinetics in SARS-CoV-2 infection. IgA, IgG, and IgM are represented. The figure describes an approximate timeline of appearance and subsequent decrease of each immunoglobulin isotype following a SARS-CoV-2 infection. The curves and values are based on recently published papers [6,78,79]. S.O. Symptom onset, W. week(s), PSO. Post-symptom onset.

IgG, the most abundant antibody isotype in human blood, drives antigen-antibody interactions to protect the host against pathogens [[68], [69], [70]]. IgG can be detected as early as four days of PSO in some diseases, but samples of individuals infected with SARS-CoV-2 might take more days [[71], [72], [73]]. Specifically, longitudinal studies assessing SARS-CoV-2 IgG response reported that IgG to S2 subunit is detectable earlier than N or RBD [74,75]. Other studies reported that IgG to N protein tends to decay earlier [76], thus only can be taken as an early marker of infection but not as a reliable marker for follow-up diagnostics [76,77]. The overall S1- and S2-IgG response peaks 50 days PSO and remains steady for up to eight months [74,75,77] (Fig. 2). To summarize, IgG titer to N-antigen tends to reflect the patient's infection status, while IgG titer to S-protein reflects the neutralization antibody titer of an individual, as previously reported [76].

IgM is generally expressed on the surface of B cells and is detectable after the first week of PSO, while it has a lower afﬁnity and specificity to antigens [80,81]. Several studies showed that the severity of SARS-CoV-2 infection impacts the timeline production/detection of IgG or IgM. Whereas in severe and critical cases of SARS-CoV-2 infection, IgM is detectable earlier than IgG, in case of mild infection or less COVID-19 severity, IgG seroconversion is prior to IgM (Fig. 2) [82,83]. IgM has a stronger immune response to S2 than RBD, suggesting that S2-specific IgM develops earlier and more abundantly than RBD-specific IgM [83].

IgA appears earlier than IgG and IgM in respiratory infections; therefore, it might contribute to virus neutralization in the early phase of infection [[84], [85], [86]]. Guo et al. reported a high detection of SARS-CoV-2-specific IgA as higher as 92.7 % in the first week of infection, followed by IgM (85.4 %) and IgG (77.9 %), respectively [87]. A clinical survey indicated similar trends that the IgA kit showed the highest diagnostic accuracy (88.2 %) in four to ten days PSO, while IgM and IgG kits exhibited 76.4 % and 64.7 %, respectively [6]. The IgA kit harbored the highest sensitivity throughout 4–25 days after clinical symptoms. The median relative light units (RLU) of RBD-specific IgA reached to the highest level throughout 16–20 days after clinical symptoms and then decreased but at comparatively high reading levels until 31–41 days [82,83].

This high titer of IgA right after the onset suggests a longer incubation time of asymptomatic period during SARS-CoV-2 mucosal infection that coincides with and supports the early detection of IgA PSO [84,88]. IgA is not only produced earlier than IgG and IgM, but more importantly, its response against N, S1, RBD, or S2 is the same, regardless of the severity of infection, so IgA to N, S1, RBD, or S2 can be detectable simultaneously [83,89].

Using RBD-specific IgA in respiratory mucus, which can be evaluated directly from salivary and tear samples non-invasive and painless, the host immune response can be assessed to help develop vaccines against SARS CoV-2 [16]. Moreover, the superlative samples for an optimal IgA-based detection for an early SARS-CoV-2 diagnosis should be saliva and tear [16], nasopharyngeal swabs, and oropharyngeal swabs [90].

Numerous studies demonstrated the early production of IgA and its presence in symptomatic and asymptomatic suspected COVID-19 patients, making IgA the ideal immunological biomarker to determine SARS-CoV-2 in the early stage and then apply adequate measures to isolate and treat the infected patients [89,91,92].

IgA and IgG1 (coupled with IgG3) antibody response strongly correlates with SARS-CoV-2 neutralizing antibodies (nAbs), suggesting that IgA and then IgG subtypes (specifically IgG1 and IgG3) are involved in the fighting with SARS-CoV-2 infection at the very early stage [92]. These results indicate that IgA is the first line of nAb set produced by the host to protect the host against SARS-CoV-2 infection during the first week of infection [89,[91], [92], [93], [94]].

### 1.5 IgA immune response in anti-SARS-CoV-2 host defense

**Fig. 3:**
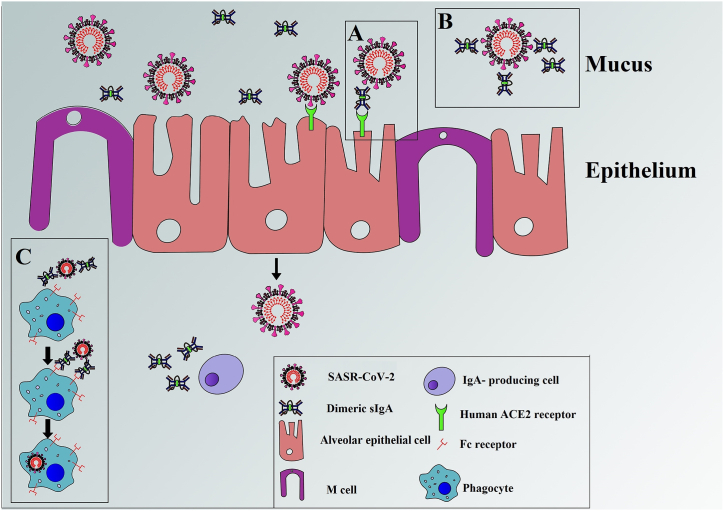
Schematic cartoon of the function of IgA in immunity (immune exclusion). A) Abrogating viral entry by blocking epithelial receptors for SARS-CoV-2. B) Entrapping viruses in mucus neutralizes SARS-CoV-2 before binding to epithelial cells. C) Targeting of myeloid dendritic cells.

SIgA via immunoglobulin receptor is introduced into the mucus, which plays several crucial functions in mucosal immunity through a stepwise series of events and contributes to immunological defense as the first line of immunity [95,96]. A side from being the quickest produced antibody, sIgA demonstrates a more specific mucosal immune response against respiratory infections, including SARS-CoV-2 stimulation [97,98]. IgA alone may trigger a strong adequate neutralizing capacity to mitigate SARS-CoV-2 infection. Primarily, sIgA are known to compete for binding to the viral ligands that trigger viral entry, preventing viral attachment and host cell infecting [99]. Moreover, IgA can bind the spike protein of SARS-CoV-2, thereby preventing the SARS-CoV-2 virus from binding to Angiotensin-converting enzyme 2 (ACE2) receptors on host cells leading to viral clearance [100]. SIgA is also functioning on myeloid immune responses through the FcαR receptor to the Fc region of IgA found on multiple immune and epithelial cells, resulting in a broad range of functions, including both humoral and cellular responses [97,98] (Fig. 3(A–C)). Several human studies indicated that immunoglobulins from the circulation are not effectively transported into external secretions. Instead, Igs in external secretions is produced locally by mucosal tissue plasma cells and not from the circulation [101]. In the effective intracellular neutralization, epithelial cells internalize IgA produced by adjacent cells with the specificity for the antigen [[102], [103], [104]]. M-cells are specialized cells located in mucosal-associated lymphoid tissue outside the gastrointestinal tract and participate in mucosal immunity by transporting antigens from the intestine lumen to mucosal lymphoid tissues, where the mucosal immune system is present [105,106]. In a study by Naito et al. a robust positive correlation between the frequency of sIgA deficiency and the high SARS-CoV-2 infection rate was observed per millions of population in patients who suffered from SARS-CoV-2 impaired production of sIgA [66]. However, strong evidence demonstrated that IgA's complete protection ability against SARS-CoV-2 infection is highly recommended.

IgA production is transient, and thus the neutralizing duration seems short, therefore; once the infection is completely installed, the IgA production deficiency is paved by the production of IgM and IgG [66,84,89,107]. A study found that later during the SARS-CoV-2 infection course, IgA negatively correlated with non-neutralizing antibodies. More specifically, the IgA titer decreased and tended to be lower than that of both IgG and binding-non-neutralizing antibodies [92]. Hypothetical involvement of non-neutralizing antibodies in antibody-dependent enhancement (ADE) diseases, IgAs are unlikely to be involved in ADE in COVID-19 severe and critical cases but rather contribute to the healing process of the infection [108,109]. In SARS-CoV-2 infections, the ADE effect has been mainly associated with hyper-production of non-neutralizing IgG antibodies or low concentration of neutralizing IgG, but not with IgA [[108], [109], [110]]. However, its role in ADE COVID-19 should be studied since Kozlowski et al. found that IgA shielded by IgG induce ADE infection [111].

### 1.6 IgA immune response in intestinal infection of SARS-CoV-2

IgA is significant in the intestinal tract and synthesized in higher quantities than other antibodies, as it protects the intestinal mucosa [[84], [85], [86],^112^]. It was dementated that IgA can bind to microorganisms, toxins, antigens, and foreign proteins to inhibit their penetration into the intestinal epithelium [113,114]. Regulation of commensal bacteria is another role of the intestinal IgA [115]. However, despite high IgA levels in the gastrointestinal tract, these roles in intestinal immunity toward viral pathogens have been challenging to be confirmed.

Scarce information is available about IgA secretion and its role in intestinal infections caused by SARS-CoV-2. Production of SARS-CoV-2-specific IgA antibody was reported in patients who suffered from SARS-CoV-2 intestinal inflammation and diarrhea [116]. IgA is the primary mucosal antibody recognized to provide long-term protection from enteric viral infections [113]. SARS-CoV-2-specific IgA has been detected in serum [117], Broncho-alveolar lavage fluid [118], nasal swabs [84], and breast milk [119] of COVID-19 positive patients. A survey on SARS-CoV-2 showed that the fecal RBD-specific IgA response is associated with serum RBD-specific IgA response. It remains to be determined whether fecal SARS-CoV-2 specific IgA represents a localized protective response within the intestines or reflects a systemic response to the viral challenge [117]. It was demonstrated a direct relationship between the increase in viral-specific IgA levels in the gastrointestinal tract with virus excretion and protection against disease [93,120,121]. Studies showed that IgA controls the initial neutralizing antibody response to SARS-CoV-2 [15,84]. Secretory IgA protects mucosal surfaces against pathogens by neutralizing viruses or impeding their attachment to intestine epithelial cells [122]. Studies demonstrated that the vaccine regimen should consider targeting a potent but potentially short-lived IgA response [14,16,123].

### 1.7 Developing mucosal vaccine inducing IgA responses

It is reported that plasmas from convalescent patients (known as convalescent plasmas - CPs) containing polyclonal antibodies capable of easing symptoms could be used to treat COVID-19 patients [13,25]. Interestingly, Zeng et al. demonstrated that in a pool of CPs, IgA is the first antibody isotype with the highest neutralizing effect and therefore provides 10 times better protection against SARS-CoV-2 infection than IgG better than IgM [25,124], supporting the hypothesis of using IgA in developing therapeutics against SARS-CoV-2 [16].

Although vaccines against SARS-CoV-2 are developed rapidly, the majorities are needle-based injectable vaccines with limitations such as handling difficulties and risk of contamination of needles, and they induce a systemic immune response that is not directly related to the location of infection. Furthermore, even though the current parenteral SARS-CoV-2 vaccination campaigns have been promising because of the production of protective immunity in vaccinated recipients, inducing a protective and effective local mucosal immunity seems suboptimal and remains questionable [125]. Like mucosal vaccines targeting respiratory tract mucosa, the needle-free vaccination directly induces a local immune response [14,16,123]. Mucosal vaccines can stimulate the production of sIgA antibodies and elicit immune protection by inducing a local mucosal immune response [126]. The mucosal vaccines require efficient adjuvants and vaccine delivery systems to penetrate the mucosal barrier and promote higher IgA titers against SARS-CoV-2 [127].

Interestingly, the optimized production of neutralizing secretory IgA antibodies, especially in naïve vaccine recipients, is obtained after a second-dose-induced boost, confirming the importance of the two-to-three doses SARS-CoV-2 vaccination program [[128], [129], [130]]. In addition, it is remarked that most studies reported that IgA antiviral immunity is mainly induced by mRNA-based vaccines than other vaccines, including attenuated vaccines [24,128,[130], [131], [132]], these mRNA vaccines added protective-value lies in their ability to induce neutralizing IgA. For instance, the currently available data demonstrated that mRNA and protein subunit vaccines over-perform better than an inactivated virus and viral-vectored vaccines in inducing humoral immunity (antibody response, specifically IgA) against wild type SARS-CoV-2 [131,133]. Therefore, strategies for the development of IgA immune response-inducing vaccines are suggested. Even though isolated IgA provided a good neutralizing effect against SARS-CoV-2 infection, IgA from a pool of SARS-CoV-2 CPs demonstrated higher efficacy, suggesting the importance of specific IgA targeting multiple and different targets sites. Therefore, developing IgA-based cocktails that target different SARS-CoV-2 antigenic sites might be promising. Moreover, developing a treatment favoring the production of SARS-CoV-2 specific IgA would be promising. SIgA is attracting more attention for future immunotherapies investigation because of its potential effect in dampening manifestations of respiratory viral infection like SARS-CoV-2 [134].

It is urged that other vaccination routes be tested to promote mucosal responses specifically. One such example is nasal delivery. It is reported that nasal delivery of an adenovirus-based vaccine bypasses pre-existing immunity to the vaccine carrier and improves the immune response in mice [135]. Indeed, Oral and intranasal Ad5 SARS-CoV-2 vaccines are effective in a golden hamster model [136]. Intranasal inoculation of SARS-CoV- in animal models caused localized virus-specific IgA secretions and subsequent immune response, providing better protection against SARS-CoV-2 than intramuscular delivery, which provides proof that mucosal-induced SARS-CoV-2 vaccine is feasible [15,137]. Furthermore, intranasal in mice vaccinations using adenovirus 5 and 19a vectored vaccines followed by systemic plasmid DNA or mRNA prime result in mucosal immunity against SARS-CoV-2 [138]. However, the experiments performed in mice may not be comparable or valid to those performed in humans.

Besides nasal and oral routes, intraperitoneal vaccination (IPV) has recently been proposed as a potential immunization route for inducing mucosal immunity in mice. However, the IPV route required certain adjuvants to induce efficient mucosal immune responses, especially the gastrointestinal and respiratory tract IgA responses. For instance, using 1,25-dihydroxyvitamin D3 as an adjuvant with an inactivated poliovirus vaccine in the IPV route induce saliva neutralizing sIgA responses in mice [139]. Likewise, IPV in pigs using *Mycoplasma hyopneumoniae* antigen adjuvanted with emulsion-oil induced an efficient serum IgG and IgA immune response specific to *M. hyopneumoniae* antigen [140]. More interestingly, IPV using an inactivated SARS-CoV-1 vaccine mixed with PIKA as a safe adjuvant-induced neutralizing IgG/IgA immune response specific to SARS-CoV-1 antigens at different mucosal areas, including in the intestine, the vaginal washes and the saliva/mouth [141]. However, the situation is different in humans, and intraperitoneal immunization with the bacterial toxoid did not stimulate mucosal immune responses [142]. Another example includes using virus-like particles (VLPs) such as SARS-CoV VLPs adjuvanted with CpG, increasing the secretion of antigen-specific IFN-γ and IL-4 in the spleen and IgA antibodies in lungs, intestine, feces, and vaginal washes [125,143], demonstrating a possible development of SARS-CoV-2 vaccine with intraperitoneal administration.

Subcutaneous and intradermal vaccine administration routes are other conventional vaccination routes used in experimental vaccines in animal models and, importantly, for human vaccine development. However, Su et al. [125] have summarized major approaches to subcutaneous and intradermal vaccine development and administration routes. Their data can be applied in developing non-invasive vaccines with robust mucosal IgA immune responses. Moreover, Mudgal et al. [144] have demonstrated a more direct approach for triggering mucosal IgA immune response straight towards epithelium activation instead of indirect activation via IgM, IgG, and then to the IgA channel. Lastly, based on current knowledge of IgA production, it is proposed vaginal and anal delivery routes that could be potential vaccine delivery routes worth exploring. CTx-B greatly enhances antigens immunogenicity when used as a fusion peptide for enhancing immunogenicity and solubility. CTx-B was previously used for dual-character vaccine candidates to make antigens more immunogenic and gain more solubility. It also acts as an excellent and viable phenotypic marker for downstream analysis via FACS, Western Blot, or Gene expression analysis via RT PCR [14,145,146].

## 2 Discussion and conclusion

Subgenus sarbecovirus is genetically classified as Betacoronavirus and is responsible for the 2003 severe acute respiratory syndrome (SARS-CoV) outbreak and the 2019 SARS-CoV-2 pandemic. The testing methods of clinical findings, chest CT and real-time PCR, are the more available approaches for diagnosis and treatment options. The high cost, less sensitivity, and time-consuming methods made it necessary to use serological tests. As early as SARS-CoV-2 infection occurs, specific antibodies are induced to be employed as a diagnostic test and subsequently an effective treatment. The efforts and research are ongoing to produce more specific drugs, including neutralizing antibodies against ACE-2, a drug to recruit the immune system, and vaccine therapy [147]. Studies have reported that baricitinib is proposed as an inhibitor of the Janus kinase (Jak-STAT) signaling pathway to inhibit ACE-2-mediated virus endocytosis [148]. Jeyanathan et al. support that mucosal pathogen vaccines administered using needles induce serum antibody production but a suboptimal protective mucosal immunity [100]. The spike protein is a potential target for developing a therapeutic option to prevent virus fusion and internalization [149]. Some evidence proposes that the immunological response and pro-inflammatory cytokines are probably underlying pathogenic mechanisms [17,150,151]. Therefore, modulation of inflammation may be helpful as adjuvant therapy [68]. IgA is one of the most abundant immunoglobulin isotypes in humans, especially produced by plasma cells in the lamina propria to protect epithelial mucous membranes from infectious agents [112]. The main protection role of IgA is completed under the IgA dimeric form, called secretory IgA, or SIgA. As the prominent function of IgA in antiviral immunity, there is an urgent need to develop novel mucosal vaccines to combat viral infections, including SARS-CoV-2. Importantly, vaccine-induced immunity is characterized by the high production of protective secretory IgA. For instance, vaccination of lactating mothers induces the production of higher titer of neutralizing IgA and IgG specific to spike protein in their blood and breast milk that protects themselves and the babies, respectively, from SARS-CoV-2 infection [128,130]. Likewise, SARS-CoV-2 vaccination induces a protective systemic and localized (respiratory mucosal) immune response characterized by the production of a very high titer of IgG and particularly IgA anti-spike neutralizing antibodies in serum, lung samples, and broncho-alveolar (upper aero digestive tract lavages of vaccine recipients) [24,152]. It has conducted a comparative study of IgA determination, the importance among immunoglobulins to combat COVID-19 infection and the protective role for the vaccination. It also functional effects, highlighting its role was demonstrated in SARS-CoV-2 infection. It is strongly recommended that further research on IgA roles as a host immunological response indicator, early detection marker, evaluation of vaccines' efficiency, and mass-producing antibodies for treatment should be performed. Lastly, the mucosal vaccine could be the future direction of vaccine design.

## Funding

This study is supported by the 10.13039/501100012166National Key Research and Development Program of China (Grants No. 2022YFC2304102), the 10.13039/501100001809National Natural Science Foundation of China (Grant No.: 82272301 and 31971129), Anhui Provincial Key Research and Development Project (Grant No. 2022i01020025), the Fundamental Research Funds for the Central Universities (WK9100000001).

## Ethics approval and consent to participate

Not applicable.

## Consent to publication

Not applicable.

## Data availability statement

There is no available data in this review article.

## CRediT authorship contribution statement

**Khaleqsefat Esmat:** Writing – original draft. **Baban Jamil:** Writing – original draft. **Ramiar Kaml Kheder:** Writing – review & editing. **Arnaud John Kombe Kombe:** Writing – original draft. **Weihong Zeng:** Supervision. **Huan Ma:** Supervision. **Tengchuan Jin:** Supervision.

## Declaration of competing interest

No competing interests to be declared.

## References

[1] Zhu N., Zhang D., Wang W., Li X., Yang B., Song J. (2020). A novel coronavirus from patients with pneumonia in China, 2019. N. Engl. J. Med..

[2] Jiang S., Shi Z.L. (2020).

[3] Afzal A. (2020 Nov). Molecular diagnostic technologies for COVID-19: limitations and challenges. J. Adv. Res..

[4] Feng W., Newbigging A.M., Le C., Pang B., Peng H., Cao Y. (2020). Molecular diagnosis of COVID-19: challenges and research needs. Anal. Chem..

[5] Bošnjak B., Odak I., Barros-Martins J., Sandrock I., Hammerschmidt S.I., Permanyer M. (2021). Intranasal delivery of MVA vector vaccine induces effective pulmonary immunity against SARS-CoV-2 in rodents. Front. Immunol..

[6] Ma H., Zeng W., He H., Zhao D., Jiang D., Zhou P. (2020). Serum IgA, IgM, and IgG responses in COVID-19. Cell. Mol. Immunol..

[7] Lippi G., Plebani A.M. SimundicM. (2020). Potential preanalytical and analytical vulnerabilities in the laboratory diagnosis of coronavirus disease 2019 (COVID-19). Clin. Chem. Lab. Med..

[8] Gong F., Wei H.X., Li Q., Li L. LiuB. (2021). Evaluation and comparison of serological methods for COVID-19 diagnosis. Front. Mol. Biosci..

[9] Pan Y., Long L., Zhang D., Yuan T., Cui S., Yang P. (2020). Potential false-negative nucleic acid testing results for severe acute respiratory syndrome coronavirus 2 from thermal inactivation of samples with low viral loads. Clin. Chem..

[10] Petherick A. (2020). Developing antibody tests for SARS-CoV-2. Lancet.

[11] Okba N.M., Müller M.A., Li W., Wang C., GeurtsvanKessel C.H., Corman V.M. (2020). Severe acute respiratory syndrome coronavirus 2− specific antibody responses in coronavirus disease patients. Emerg. Infect. Dis..

[12] Dziedzic R. Kubina A. (2020). Molecular and serological tests for COVID-19 a comparative review of SARS-CoV-2 coronavirus laboratory and point-of-care diagnostics. Diagnostics.

[13] Mantis N.J., Forbes S.J. (2010). Secretory IgA: arresting microbial pathogens at epithelial borders. Immunol. Invest..

[14] Kim M.H., Kim H.J., Chang J. (2019). Superior immune responses induced by intranasal immunization with recombinant adenovirus-based vaccine expressing full-length Spike protein of Middle East respiratory syndrome coronavirus. PLoS One.

[15] See R.H., Zakhartchouk A.N., Petric M., Lawrence D.J., Mok C.P., Hogan R.J. (2006). Comparative evaluation of two severe acute respiratory syndrome (SARS) vaccine candidates in mice challenged with SARS coronavirus. J. Gen. Virol..

[16] Chao Y.X., Tan O. RötzschkeE.-K. (2020). The role of IgA in COVID-19. Brain Behav. Immun..

[17] Li G., Fan Y., Lai Y., Han T., Li Z., Zhou P. (2020 Apr). Coronavirus infections and immune responses. J. Med. Virol..

[18] Sheikh-Mohamed S., Isho B., Chao G.Y., Zuo M., Cohen C., Lustig Y. (2022). Systemic and mucosal IgA responses are variably induced in response to SARS-CoV-2 mRNA vaccination and are associated with protection against subsequent infection. Mucosal Immunol..

[19] Vâţă A., Anita A., Manciuc C.D., Savuta G., Luca C.M., Roșu F.M. (2022). Clinical significance of early IgA anti-SARS-CoV-2 antibody detection in patients from a Romanian referral COVID-19 hospital. Exp. Ther. Med..

[20] Jearanaiwitayakul T., Seesen M., Chawengkirttikul R., Limthongkul J., Apichirapokey S., Sapsutthipas S. (2021). Intranasal administration of RBD nanoparticles confers induction of mucosal and systemic immunity against SARS-CoV-2. Vaccines.

[21] Muramatsu M., Yoshida R., Yokoyama A., Miyamoto H., Kajihara M., Maruyama J. (2014). Comparison of antiviral activity between IgA and IgG specific to influenza virus hemagglutinin: increased potential of IgA for heterosubtypic immunity. PLoS One.

[22] Ejemel M., Li Q., Hou S., Schiller Z.A., Wallace A.L., Amcheslavsky A. (2020).

[23] Ejemel M., Li Q., Hou S., Schiller Z.A., Tree J.A., Wallace A. (2020). A cross-reactive human IgA monoclonal antibody blocks SARS-CoV-2 spike-ACE2 interaction. Nat. Commun..

[24] Jearanaiwitayakul T., Apichirapokey S., Chawengkirttikul R., Limthongkul J., Seesen M., Jakaew P. (2021). Peritoneal administration of a subunit vaccine encapsulated in a nanodelivery system not only augments systemic responses against SARS-CoV-2 but also stimulates responses in the respiratory tract. Viruses.

[25] Zeng W., Ma H., Ding C., Yang Y., Sun Y., Huang X. (2021). Characterization of SARS-CoV-2-specific antibodies in COVID-19 patients reveals highly potent neutralizing IgA. Signal Transduct. Targeted Ther..

[26] Mu S., Song S., Hao Y., Luo F., Wu R., Wang Y. (2022). Neutralizing antibodies from the rare convalescent donors elicited antibody-dependent enhancement of SARS-CoV-2 variants infection. Front. Med..

[27] Clark N.M., Janaka S.K., Hartman W., Stramer S., Goodhue E., Weiss J. (2022). Anti-SARS-CoV-2 IgG and IgA antibodies in COVID-19 convalescent plasma do not enhance viral infection. PLoS One.

[28] Zervou F.N., Louie P., Stachel A., Zacharioudakis I.M., Ortiz‐Mendez Y., Thomas K. (2021). SARS‐CoV‐2 antibodies: IgA correlates with severity of disease in early COVID‐19 infection. J. Med. Virol..

[29] Americo J.L., Cotter C.A., Earl P.L., Moss R. LiuB. (2022). Intranasal inoculation of an MVA-based vaccine induces IgA and protects the respiratory tract of hACE2 mice from SARS-CoV-2 infection. Proc. Natl. Acad. Sci. USA.

[30] Müller M., Volzke J., Subin B., Müller S., Sombetzki M., Reisinger E.C. (2022). Single-dose SARS-CoV-2 vaccinations with either BNT162b2 or AZD1222 induce disparate Th1 responses and IgA production. BMC Med..

[31] Woof J.M., Kerr M.A. (2004). IgA function--variations on a theme. Immunology.

[32] de Sousa-Pereira P., Woof J.M. (2019). IgA: structure, function, and developability. Antibodies.

[33] Woof J., Russell M. (2011). Structure and function relationships in IgA. Mucosal Immunol..

[34] Ruprecht R.M., Thippeshappa B. MarasiniR. (2019). Mucosal antibodies: defending epithelial barriers against HIV-1 invasion. Vaccines (Basel).

[35] Snoeck V., Cox I.R. PetersE. (2006). The IgA system: a comparison of structure and function in different species. Vet. Res..

[36] Johansen F.-E., Brandtzaeg R. BraathenP. (2001). The J chain is essential for polymeric Ig receptor-mediated epithelial transport of IgA. J. Immunol..

[37] Halpern M.S., Koshland M.E. (1970). Novel subunit in secretory IgA. Nature.

[38] Johansen F.E., Brandtzaeg R. BraathenP. (2000). Role of J chain in secretory immunoglobulin formation. Scand. J. Immunol..

[39] Leong K., Ding J. (2014). The unexplored roles of human serum IgA. DNA Cell Biol..

[40] Phalipon A., Cardona A., Kraehenbuhl J.P., Edelman L., Corthesy P.J. SansonettiB. (2002). Secretory component: a new role in secretory IgA-mediated immune exclusion in vivo. Immunity.

[41] Fallgren-Gebauer E., Gebauer W., Bastian A., Kratzin H., Eiffert H., Zimmermann B. (1995). Advances in Mucosal Immunology.

[42] Tomasi T.B., Tan E.M., SolomonR A., Prendergast A. (1965). Characteristics of an immune system common to certain external secretions. J. Exp. Med..

[43] Kaetzel C.S. (2005). The polymeric immunoglobulin receptor: bridging innate and adaptive immune responses at mucosal surfaces. Immunol. Rev..

[44] Frutiger S., Hughes G.J., Hanly W., Jaton M. KingzetteJ.-C. (1986). The amino-terminal domain of rabbit secretory component is responsible for noncovalent binding to immunoglobulin A dimers. J. Biol. Chem..

[45] Stadtmueller B.M., Huey-Tubman K.E., López C.J., Yang Z., HubbellP W.L., Bjorkman J. (2016). The structure and dynamics of secretory component and its interactions with polymeric immunoglobulins. Elife.

[46] Kumar Bharathkar S., Parker B.W., Malyutin A.G., Haloi N., Huey-Tubman K.E., Tajkhorshid E. (2020). The structures of secretory and dimeric immunoglobulin A. Elife.

[47] Kumar N., Arthur C.P., CiferriM C., Matsumoto L. (2020). Structure of the secretory immunoglobulin A core. Science.

[48] Jertborn M., Holmgren A. SvennerholmJ. (1986). Saliva, breast milk, and serum antibody responses as indirect measures of intestinal immunity after oral cholera vaccination or natural disease. J. Clin. Microbiol..

[49] Dimmock S. Armstrong N. (1992). Neutralization of influenza virus by low concentrations of hemagglutinin-specific polymeric immunoglobulin A inhibits viral fusion activity, but activation of the ribonucleoprotein is also inhibited. J. Virol..

[50] Mazanec M.B., CoudretD C.L., Fletcher R. (1995). Intracellular neutralization of influenza virus by immunoglobulin A anti-hemagglutinin monoclonal antibodies. J. Virol..

[51] Mazanec M.B., Kaetzel C.S., Lamm M.E., FletcherJ D., Nedrud G. (1992). Intracellular neutralization of virus by immunoglobulin A antibodies. Proc. Natl. Acad. Sci. USA.

[52] Burns J.W., Siadat-Pajouh M., KrishnaneyH A.A., Greenberg B. (1996). Protective effect of rotavirus VP6-specific IgA monoclonal antibodies that lack neutralizing activity. Science.

[53] Havervall S., Marking U., Svensson J., Greilert-Norin N., Bacchus P., Nilsson P. (2022). Anti-spike mucosal IgA protection against SARS-CoV-2 omicron infection. N. Engl. J. Med..

[54] Wang Z., Lorenzi J.C., Muecksch F., Finkin S., Viant C., Gaebler C. (2020).

[55] Kaetzel C.S., Robinson J.K., Chintalacharuvu K.R., VaermanM J.-P., Lamm E. (1991). The polymeric immunoglobulin receptor (secretory component) mediates transport of immune complexes across epithelial cells: a local defense function for IgA. Proc. Natl. Acad. Sci. USA.

[56] Robinson J.K., Blanchard T.G., Levine A.D., EmancipatorM S.N., Lamm E. (2001). A mucosal IgA-mediated excretory immune system in vivo. J. Immunol..

[57] Gan Y., Chodosh J., MorganJ A., Sixbey W. (1997). Epithelial cell polarization is a determinant in the infectious outcome of immunoglobulin A-mediated entry by Epstein-Barr virus. J. Virol..

[58] Bomsel M., Heyman M., Hocini H., Lagaye S., Belec L., Dupont C. (1998). Intracellular neutralization of HIV transcytosis across tight epithelial barriers by anti-HIV envelope protein dIgA or IgM. Immunity.

[59] Chia W.N., Tan C.W., Foo R., Kang A.E.Z., Peng Y., Sivalingam V. (2020). Serological differentiation between COVID-19 and SARS infections. Emerg. Microb. Infect..

[60] Tang Y.-W., Schmitz J.E., PersingC D.H., Stratton W. (2020). Laboratory diagnosis of COVID-19: current issues and challenges. J. Clin. Microbiol..

[61] Lal M. Surjit S. (2008). The SARS-CoV nucleocapsid protein: a protein with multifarious activities. Infect. Genet. Evol..

[62] Burbelo P.D., Riedo F.X., Morishima C., Rawlings S., Smith D., Das S. (2020).

[63] Jacot D., Moraz M., Coste A.T., Aubry C., Sacks J.A., Greub G. (2021). Evaluation of sixteen ELISA SARS-CoV-2 serological tests. J. Clin. Virol..

[64] Cervia C., Nilsson J., Zurbuchen Y., Valaperti A., Schreiner J., Wolfensberger A. (2021). Systemic and mucosal antibody responses specific to SARS-CoV-2 during mild versus severe COVID-19. J. Allergy Clin. Immunol..

[65] Li N., Wang P., Wang X., Geng C., Gong J. ChenY. (2020). Molecular diagnosis of COVID-19: current situation and trend in China. Exp. Ther. Med..

[66] Naito Y., Takagi T., Watanabe T. YamamotoS. (2020). Association between selective IgA deficiency and COVID-19. J. Clin. Biochem. Nutr..

[67] Schroeder H.W., Cavacini L. (2010). Structure and function of immunoglobulins. J. Allergy Clin. Immunol..

[68] Ghaffari A., Ardakani R. MeurantA. (2020). COVID-19 serological tests: how well do they actually perform?. Diagnostics.

[69] Jacofsky D., Jacofsky E.M. JacofskyM. (2020). Understanding antibody testing for COVID-19. J. Arthroplasty.

[70] de Taeye S.W., Vidarsson T. RispensG. (2019). The ligands for human IgG and their effector functions. Antibodies.

[71] Rashid Z.Z., Othman S.N., Samat M.N.A., AliK U.K., Wong K. (2020). Diagnostic performance of COVID-19 serology assays. Malays. J. Pathol..

[72] Sun B., Feng Y., Mo X., Zheng P., Wang Q., Li P. (2020). Kinetics of SARS-CoV-2 specific IgM and IgG responses in COVID-19 patients. Emerg. Microb. Infect..

[73] Hou H., Wang T., Zhang B., Luo Y., Mao L., Wang F. (2020). Detection of IgM and IgG antibodies in patients with coronavirus disease 2019. Clinical & translational immunology.

[74] Shi D., Weng T., Wu J., Dai C., Luo R., Chen K. (2021). Dynamic characteristic analysis of antibodies in patients with COVID-19: a 13-month study. Front. Immunol..

[75] Liao B., Chen Z., Zheng P., Li L., Zhuo J., Li F. (2021). Detection of anti-SARS-CoV-2-S2 IgG is more sensitive than anti-RBD IgG in identifying asymptomatic COVID-19 patients. Front. Immunol..

[76] Fujigaki H., Inaba M., Osawa M., Moriyama S., Takahashi Y., Suzuki T. (2021). Comparative analysis of antigen-specific anti-SARS-CoV-2 antibody isotypes in COVID-19 patients. J. Immunol..

[77] Orth-Holler D., Eigentler A., Stiasny K., WeseslindtnerJ L. (2021). Most, Kinetics of SARS-CoV-2 specific antibodies (IgM, IgA, IgG) in non-hospitalized patients four months following infection. J. Infect..

[78] Pang N.Y.L., Pang A.S.R., ChowD V.T., Wang Y. (2021). Understanding neutralising antibodies against SARS-CoV-2 and their implications in clinical practice. Military Medical Research.

[79] Galipeau Y., Greig M., Liu G., DriedgerM M., Langlois A. (2020). Humoral responses and serological assays in SARS-CoV-2 infections. Front. Immunol..

[80] Gong S., Ruprecht R.M. (1943). Immunoglobulin M: An ancient antiviral weapon–rediscovered. Front. Immunol..

[81] Lou B., Li T.-D., Zheng S.-F., Su Y.-Y., Li Z.-Y., Liu W. (2020). Serology characteristics of SARS-CoV-2 infection after exposure and post-symptom onset. Eur. Respir. J..

[82] Long Q.X., Liu B.Z., Deng H.J., Wu G.C., Deng K., Chen Y.K. (2020). Antibody responses to SARS-CoV-2 in patients with COVID-19. Nat. Med..

[83] Jamiruddin M.R., Haq M.A., Tomizawa K., Kobatake E., Mie M., Ahmed S. (2021). Longitudinal antibody dynamics against structural proteins of SARS-CoV-2 in three COVID-19 patients shows concurrent development of IgA, IgM, and IgG. J. Inflamm. Res..

[84] Sterlin D., Mathian A., Miyara M., Mohr A., Anna F., Claër L. (2021). IgA dominates the early neutralizing antibody response to SARS-CoV-2. Sci. Transl. Med..

[85] Boes M. (2000). Role of natural and immune IgM antibodies in immune responses. Mol. Immunol..

[86] Renegar K.B., Small P.A., BoykinsP L.G., Wright F. (2004). Role of IgA versus IgG in the control of influenza viral infection in the murine respiratory tract. J. Immunol..

[87] Guo L., Ren L., Yang S., Xiao M., Chang, Yang F. (2020). Profiling early humoral response to diagnose novel coronavirus disease (COVID-19). Clin. Infect. Dis..

[88] Cervia C., Nilsson J., Zurbuchen Y., Valaperti A., Schreiner J., Wolfensberger A. (2021). Systemic and mucosal antibody responses specific to SARS-CoV-2 during mild versus severe COVID-19. J. Allergy Clin. Immunol..

[89] Cravedi P., Ahearn P., Wang L., Yalamarti T., Hartzell S., Azzi Y. (2021).

[90] Lee R.A., Herigon J.C., Benedetti A., PollockC N.R., Denkinger M. (2021). Performance of saliva, oropharyngeal swabs, and nasal swabs for SARS-CoV-2 molecular detection: a systematic review and meta-analysis. J. Clin. Microbiol..

[91] Brandtzaeg P. (2013). Secretory IgA: designed for anti-microbial defense. Front. Immunol..

[92] Patil H.P., Rane P.S., Shrivastava S., Palkar S., Lalwani S., Mishra A.C. (2021). Antibody (IgA, IgG, and IgG subtype) responses to SARS-CoV-2 in severe and nonsevere COVID-19 patients. Viral Immunol..

[93] Blutt S.E., Miller A.D., Salmon S.L., MetzgerM D.W., Conner E. (2012). IgA is important for clearance and critical for protection from rotavirus infection. Mucosal Immunol..

[94] Macpherson A., McCoy K., Brandtzaeg F. JohansenP. (2008). The immune geography of IgA induction and function. Mucosal Immunol..

[95] Vidarsson R. Horton G. (2013). Antibodies and their receptors: different potential roles in mucosal defense. Front. Immunol..

[96] Frey A., Lunding L.P., Ehlers J.C., Weckmann M., Wegmann U.M. ZisslerM. (2020). More than just a barrier: the immune functions of the airway epithelium in asthma pathogenesis. Front. Immunol..

[97] Breedveld A., Van Egmond M., IgA and FcαRI (2019). Pathological roles and therapeutic opportunities. Front. Immunol..

[98] Varadhachary A., Chatterjee D., Garza J., Garr R.P., Foley C., Letkeman A.F. (2020).

[99] Corthesy B. (2013). Role of secretory IgA in infection and maintenance of homeostasis. Autoimmun. Rev..

[100] Jeyanathan M., Afkhami S., Smaill F., Miller M.S., Xing B.D. LichtyZ. (2020). Immunological considerations for COVID-19 vaccine strategies. Nat. Rev. Immunol..

[101] Mestecky J. (2021).

[102] Mantis N.J., Corthesy N. RolB. (2011). Secretory IgA's complex roles in immunity and mucosal homeostasis in the gut. Mucosal Immunol..

[103] Forthal D.N. (2014). Functions of antibodies. Microbiol. Spectr..

[104] Corthesy B. (2013). Multi-faceted functions of secretory IgA at mucosal surfaces. Front. Immunol..

[105] Lo A. Dillon D.D. (2019). M cells: intelligent engineering of mucosal immune surveillance. Front. Immunol..

[106] Corr S.C., Hill C.C. GahanC. (2008). M-cells: origin, morphology and role in mucosal immunity and microbial pathogenesis. FEMS Immunol. Med. Microbiol..

[107] Monsell E.M., Cody D.D., SpicklerJ E., Windham P. (1997). Segmentation of acoustic neuromas with magnetic resonance imaging and Eigen image filtering. Am. J. Otol..

[108] Wang S.F., Tseng S.P., Yen C.H., Yang J.Y., Tsao C.H., Shen C.W. (2014). Antibody-dependent SARS coronavirus infection is mediated by antibodies against spike proteins. Biochem. Biophys. Res. Commun..

[109] Francesco N. (2020). Is antibody-dependent enhancement playing a role in COVID-19 pathogenesis?. Swiss Med. Wkly..

[110] Kombe Kombe A.J., Zahid A., Mohammed A., Jin R. ShiT. (2021). Potent molecular feature-based neutralizing monoclonal antibodies as promising therapeutics against SARS-CoV-2 infection. Front. Mol. Biosci..

[111] Kozlowski P.A., Black K.P., Jackson L. ShenS. (1995). High prevalence of serum IgA HIV-1 infection-enhancing antibodies in HIV-infected persons. Masking by IgG. J. Immunol..

[112] Slack O. Pabst E. (2020). IgA and the intestinal microbiota: the importance of being specific. Mucosal Immunol..

[113] Pietrzak B., Tomela K., Olejnik-Schmidt A., Schmidt A. MackiewiczM. (2020). Secretory IgA in intestinal mucosal secretions as an adaptive barrier against microbial cells. Int. J. Mol. Sci..

[114] Corthésy B. (2013). Multi-faceted functions of secretory IgA at mucosal surfaces. Front. Immunol..

[115] Mathias A., Pais B., Favre L., Corthésy J. BenyacoubB. (2014). Role of secretory IgA in the mucosal sensing of commensal bacteria. Gut Microb..

[116] Britton G.J., Chen-Liaw A., Cossarini F., Livanos A.E., Spindler M.P., Plitt T. (2020).

[117] Conner M.E., Blutt S.E. (2013). The gastrointestinal frontier: IgA and viruses. Front. Immunol..

[118] Amanat F., Stadlbauer D., Strohmeier S., Nguyen T.H., Chromikova V., McMahon M. (2020). A serological assay to detect SARS-CoV-2 seroconversion in humans. Nat. Med..

[119] Cervia C., Nilsson J., Zurbuchen Y., Valaperti A., Schreiner J., Wolfensberger A. (2020).

[120] Fox A., Marino J., Amanat F., Krammer F., Hahn-Holbrook J., Zolla-Pazner S. (2020).

[121] Alvarez N., Sarmiento M.E., Acosta N. Mohd-NorA. (2014). Uses of immunoglobulin A in the control of the infectious diseases. Biotecnol. Apl..

[122] Padoan A., Sciacovelli L., Basso D., Negrini D., Zuin S., Cosma C. (2020). IgA-Ab response to spike glycoprotein of SARS-CoV-2 in patients with COVID-19: a longitudinal study. Clin. Chim. Acta.

[123] Russell M.W., Moldoveanu Z., Ogra P.L., Mestecky J. (2020). Mucosal immunity in COVID-19: a neglected but critical aspect of SARS-CoV-2 infection. Front. Immunol..

[124] Sterlin D., Mathian A., Miyara M., Mohr A., Anna F., Claer L. (2021). IgA dominates the early neutralizing antibody response to SARS-CoV-2. Sci. Transl. Med..

[125] Su F., Patel G.B., Chen S. HuW. (2016). Induction of mucosal immunity through systemic immunization: phantom or reality?. Hum. Vaccines Immunother..

[126] Cerutti K. Chen A. (2010). Vaccination strategies to promote mucosal antibody responses. Immunity.

[127] Boyaka P.N. (2017). Inducing mucosal IgA: a challenge for vaccine adjuvants and delivery systems. J. Immunol..

[128] Juncker H.G., Mulleners S.J., van Gils M.J., de Groot C.J.M., Pajkrt D., Korosi A. (2021). The levels of SARS-CoV-2 specific antibodies in human milk following vaccination. J. Hum. Lactation.

[129] Singhatiraj E., Pongpirul K., Hirankarn A. JongkaewwattanaN. (2021). Intradermal ChAdOx1 vaccine following two CoronaVac shots: a case report. Vaccines (Basel)..

[130] Goncalves J., Juliano A.M., Charepe N., Alenquer M., Athayde D., Ferreira F. (2021). Secretory IgA and T cells targeting SARS-CoV-2 spike protein are transferred to the breastmilk upon mRNA vaccination. Cell Rep Med.

[131] Wisnewski A.V., Redlich J. Campillo LunaC.A. (2021). Human IgG and IgA responses to COVID-19 mRNA vaccines. PLoS One.

[132] Chan R.W.Y., Liu S., Cheung J.Y., Tsun J.G.S., Chan K.C., Chan K.Y.Y. (2021). The mucosal and serological immune responses to the novel coronavirus (SARS-CoV-2) vaccines. Front. Immunol..

[133] Souza W.M., Amorim M.R., Sesti-Costa R., Coimbra L.D., Brunetti N.S., Toledo-Teixeira D.A. (2021). Neutralisation of SARS-CoV-2 lineage P.1 by antibodies elicited through natural SARS-CoV-2 infection or vaccination with an inactivated SARS-CoV-2 vaccine: an immunological study. Lancet Microbe.

[134] Matuchansky C. (2021). Mucosal immunity to SARS-CoV-2: a clinically relevant key to deciphering natural and vaccine-induced defences. Clin. Microbiol. Infect..

[135] Croyle M.A., Patel A., Tran K.N., Gray M., Zhang Y., Strong J.E. (2008). Nasal delivery of an adenovirus-based vaccine bypasses pre-existing immunity to the vaccine carrier and improves the immune response in mice. PLoS One.

[136] Langel S.N., Johnson S., Martinez C.I., Tedjakusuma S.N., Peinovich N., Dora E.G. (2021).

[137] Chavda V.P., Vora L.K., PandyaV A.K., Patravale B. (2021). Intranasal vaccines for SARS-CoV-2: from challenges to potential in COVID-19 management. Drug Discov. Today.

[138] Lapuente D., Fuchs J., Willar J., Antao A.V., Eberlein V., Uhlig N. (2021). Protective mucosal immunity against SARS-CoV-2 after heterologous systemic prime-mucosal boost immunization. Nat. Commun..

[139] Ivanov A.P., DragunskyK E.M., Chumakov M. (2006). 1,25-dihydroxyvitamin d3 enhances systemic and mucosal immune responses to inactivated poliovirus vaccine in mice. J. Infect. Dis..

[140] Sheldrake R.F., RomalisM L.F., Saunders M. (1993). Serum and mucosal antibody responses against Mycoplasma hyopneumoniae following intraperitoneal vaccination and challenge of pigs with M hyopneumoniae. Res. Vet. Sci..

[141] Gai W.W., Zhang Y., Zhou D.H., Chen Y.Q., YangH J.Y., Yan M. (2011). PIKA provides an adjuvant effect to induce strong mucosal and systemic humoral immunity against SARS-CoV. Virol. Sin..

[142] Grangette C., Muller-Alouf H., Goudercourt D., Geoffroy M.C., Mercenier M. TurneerA. (2001). Mucosal immune responses and protection against tetanus toxin after intranasal immunization with recombinant Lactobacillus plantarum. Infect. Immun..

[143] Lu B., Huang Y., Huang L., Li B., Zheng Z., Chen Z. (2010). Effect of mucosal and systemic immunization with virus-like particles of severe acute respiratory syndrome coronavirus in mice. Immunology.

[144] Mudgal R., Tomar S. NehulS. (2020). Prospects for mucosal vaccine: shutting the door on SARS-CoV-2. Hum. Vaccines Immunother..

[145] Baldauf K.J., Royal J.M., Matoba K.T. HamorskyN. (2015). Cholera toxin B: one subunit with many pharmaceutical applications. Toxins.

[146] Asadi Karam M.R., Bouzari M. HabibiS. (2016). Use of flagellin and cholera toxin as adjuvants in intranasal vaccination of mice to enhance protective immune responses against uropathogenic Escherichia coli antigens. Biologicals.

[147] Kruse R.L. (2020). Therapeutic strategies in an outbreak scenario to treat the novel coronavirus originating in Wuhan, China. F1000Res..

[148] Richardson P., Griffin I., Tucker C., Smith D., Oechsle O., Phelan A. (2020). Baricitinib as potential treatment for 2019-nCoV acute respiratory disease. Lancet.

[149] Xia S., Zhu Y., Liu M., Lan Q., Xu W., Wu Y. (2020).

[150] Chen L., Liu H.G., Liu W., Liu J., Liu K., Shang J. (2020). [Analysis of clinical features of 29 patients with 2019 novel coronavirus pneumonia]. Zhonghua Jiehe He Huxi Zazhi.

[151] Huang C., Wang Y., Li X., Ren L., Zhao J., Hu Y. (2020).

[152] Bosnjak B., Odak I., Barros-Martins J., Sandrock I., Hammerschmidt S.I., Permanyer M. (2021). Intranasal delivery of MVA vector vaccine induces effective pulmonary immunity against SARS-CoV-2 in rodents. Front. Immunol..

